# Th17 cytokines regulate profibrotic cytokines release by human eosinophils

**DOI:** 10.1186/1710-1492-8-S1-A2

**Published:** 2012-11-02

**Authors:** Rabih Halwani, Saleh Al Muhsen, Hamdan Al Jahdali, Qutayba Hamid

**Affiliations:** 1 Asthma Research Chair and Prince Naif Center for Immunology Research, College of Medicine and Department of Pediatrics, King Saud University, Riyadh, Saudi Arabia; 2 King Saud University for Health Sciences, Internal Medicine, Riyadh, Saudi Arabia; 3 Meakins-Christie Laboratories, McGill University, Montreal, Canada

## Rationale

Asthma is a chronic inflammatory disorder of the lung airways that is associated with airway remodeling and hyperresponsiveness. One of the most critical structural changes that affect airway functionality is fibrotic tissue deposition within the airway wall. Eosinophils have been proposed in different studies to contribute to the production of several mediators and cytokines, including the profibrotic cytokines, TGF-β and IL-11. In this study, we hypothesize that cytokines prevailing in asthmatic tissue such as Th1, Th2, and Th17 cytokines, may induce eosinophils to produce pro-fibrotic cytokines.

## Methods

Eosinophils were isolated from peripheral blood of 6 mild asthmatics and 6 normal control subjects. Eosinophils were stimulated with Th1, Th2 and Th17 cytokines and production of pro-fibrotic cytokines, TGF-β and IL-11, were determined using Intra-cellular cytokine detection and FACS analysis, immunohistochemistry, as well as real time PCR.

## Results

The level of basal expression of eosinophil TGF-β and IL-11 was significantly upregulated in asthmatic patients compared to healthy individuals. Stimulating eosinophils with Th1 and Th2 cytokines did not induce expression of eosinophils derived pro-fibrotic cytokines. However, stimulating eosinophils with IL-17 resulted in the enhancement of the expression TGF-β and IL-11 in asthmatic individuals.

## Conclusions

The regulation of expression of pro-fibrotic cytokines within eosinophils is Th1/Th2 independent. However, IL-17 seems to regulate eosinophl profibrotic cytokine release in asthmatic patients and hence contributing to the accumulation of fibrotic tissue in asthmatic airways.

